# Gray Matter Differences Between Premature Pubertal Girls With and Without the Reactivation of the Hypothalamic—Pituitary-Gonadal Axis

**DOI:** 10.3389/fpsyt.2020.00784

**Published:** 2020-08-11

**Authors:** Yuchuan Fu, Wenjing Zhang, Bo Tao, Beisheng Yang, Di Yang, Xiaoling Xie, Peining Liu, Yaxin Zhu, Lu Zhou, Tao Chen, Xiaozheng Liu, Zhihan Yan

**Affiliations:** ^1^ Department of Radiology, Second Affiliated Hospital and Yuying Children’s Hospital of Wenzhou Medical University, Wenzhou, China; ^2^ Department of Radiology, West China Hospital of Sichuan University, Chengdu, China; ^3^ Department of Radiology, Zhejiang Hospital, Hangzhou, China; ^4^ Department of Child Health Care, The Second Affiliated Hospital and Yuying Children’s Hospital of Wenzhou Medical University, Wenzhou, China

**Keywords:** cortical thickness, follicle stimulating hormone, luteinizing hormone, estradiol, hypothalamic-pituitary-gonadal axis

## Abstract

The onset of puberty and related hormones exerts significant effects on brain morphometric and psychosocial development. The biological mechanisms underlying how the reactivation of the hypothalamic-pituitary-gonadal (HPG) axis and puberty-related hormonal maturation sculpts human brain architecture remain elusive. To address this question, 105 premature pubertal girls (age 8–11 years) without menstruation underwent brain structural scanning on a 3T MR system, and the luteinizing hormone releasing hormone (LHRH) stimulation test was used to identify the reactivation of the HPG axis. Among the 105 girls, 63 were positive for HPG axis reactivation (HPG+), while the others showed negative (HPG-). Cortical thickness was calculated and compared between the two groups after adjusting for age. The brain regions showing inter-group differences were then extracted and correlated with the peak value of serum hormone after the LHRH stimulation test in entire sample. Compared to HPG- girls, HPG+ girls showed reduced cortical thickness mainly in the the right precuneus, right inferior temporal gyrus, and right superior frontal gyrus, while increased cortical thickness primarily in the left superior parietal lobe and right inferior parietal lobe. Linear-regression analysis revealed negative correlations between the cortical thickness of the right inferior parietal lobe with the peak value of FSH and the right precuneus with LH and E. These findings provide evidence to support the notion that the reactivation of HPG axis and changes of hormones during the early phase of hormonal maturation exert influences on the development of gray matter.

## Introduction

Puberty is a critical period in which dramatic hormone-related changes parallel changes in emotion, behavior, cognition, physical, and brain development (1, 2). Notably, the effect of puberty on psychosocial development in adolescence is believed to vary at different stages, with the early phase of puberty appearing to play a more critical role than those in middle and late adolescence (3). The onset of puberty is often deemed as a trigger of several neuroendocrine-related changes, such as a vast increase in sexual hormone, which occur prior to the physical changes. The released gonadotropin releasing hormone (GnRH) is distributed in the hypothalamus in a pulsatile fashion and initiates a cascade of events that have been recognized as the onset of puberty. Following the secretion of GnRH, the pituitary responds by secreting luteinizing hormone (LH) and follicular stimulating hormone (FSH). These major hormonal changes occur just after the reactivation of the hypothalamic-pituitary-gonadal (HPG) axis, causing gonadal maturation and the production of sex steroids, most notably estrogen (E) and testosterone (TES). The first activation of the HPG axis begins at the prenatal and the early postnatal months. After the first year of birth, the HPG axis lays dormant until the resurrection of the GnRH, which facilitate the onset of puberty (4). The increased secretion of LH from the pituitary gland is the first measurable endocrinological marker of puberty (5). Generally, a serum LH level of ≥5 IU/L using modern ultrasensitive automated chemiluminescence assays after the luteinizing hormone releasing hormone (LHRH) stimulation test from patient samples (either 30 or 60 min) can be recognized as being positive for HPG axis reactivation, otherwise being negative for HPG axis reactivation following previously established clinical criteria (6–9). In the occasional child whose LH is not quite as high, they showed the auxological and clinical signs of puberty, a LHRH stimulation test may be required to confirm the diagnosis. However, sexual maturation in the previous research was defined by using biological features or clinical features [i.e., Tanner stage (10, 11) or the self-report of children (12)]. Therefore, it is necessary to specify the brain regions on which the HPG axis reactivation showed association in early adolescence.

Previous studies have found that brain morphology differs at different stages of puberty. A few studies found that the pubertal stage, as assessed by Tanner staging of sexual maturation, is related to brain morphology. For instance, girls in mid/late puberty had signiﬁcantly lower right and left cortical gray matter volumes than girls in earlier stages of puberty (13). The gray matter volume of inferior frontal gyrus in adolescents was larger in early pubertal stages compared to that in late puberty (12). The volume in the right hippocampus decreased with increasing Tanner hair stage, while the volume of the right and left amygdala decreased with increasing Tanner breast stage (14). In addition, greater increases in Tanner stage predicted less cortical thickness in the right superior frontal gyrus and right superior temporal gyrus and decreases in the left precuneus surface area in the girls (15). Goddings and her colleagues also demonstrated that the volume of the amygdala and hippocampus increased and the volume of nucleus accumbens, caudate, putamen, and globus pallidus decreased across puberty as measured by Tanner stage (16). While in a large group of 9-year-old twins, including males, the children that showed signs of physical maturation (Tanner stage ≥ 2) was associated with decreased parietal gray matter densities compared to the children without any secondary pubertal signs (Tanner stage ≤ 1) (17). However, how brain development is regulated by pubertal changes, especially at the early stage premenarche, remains poorly understood (14). Previous studies either in animals or humans found that puberty-related hormones were associated with the development of the gray matter in brain. In animal models, puberty-related hormonal changes have been shown to induce changes in brain maturation, including structural reorganization and neuronal circuit pruning, with these effects starting at puberty onset and continuing through adolescence (18–20). Several studies in humans have been conducted to examine the associations between changes in serum hormones and brain structure during adolescence, and the findings were inconsistent. One study examined the influence of sex steroids on structural brain maturation and found that there were no effects of sex steroids on cortical thickness in adolescents aged 8–25 years (12). One study demonstrated that higher TES levels were associated with thinner left calcarine sulcus and right lingual gyrus cortex in girls (21). Another study demonstrated that higher E levels, but not TES, were associated with smaller gray matter density in prefrontal, parietal, and middle temporal areas and greater gray matter density in the middle frontal, inferior temporal, and middle occipital gyri in girls between 10 and 15 years of age (22). Another two studies also showed that changes in the TES and E levels were associated with white matter, gray matter, right amygdala, and bilateral caudate volumes, cortical thickness and surface area (15, 23). Higher FSH levels have also been associated with greater gray matter density in left prefrontal areas, hippocampus, anterior cingulate, precuneus, and right cerebellum among girls aged 9 to 12 years (24). Previous studies did not find an association between LH and gray matter (12, 24, 25); however, a trend for negative association was been suggested between the gray matter density of temporal areas and the LH in the 9-year-old twins (25). Nonetheless, these studies indicated that puberty-related hormones exerted positive or/and negative influences on gray matter structural development and played different roles in brain areas. Due to variable findings in previous studies and the importance of studying gonadotropin concentration at an early stage of hormonal maturation, more research is needed to identify hormone-related influences on brain organization during the initiation of puberty.

To achieve this aim, we enrolled girls aged 8–11 years with thelarche as participants whose bone age and uterine volume were advanced than their chronologic age, seeking to identify the influences of the HPG axis reactivation on brain organization during the initiation of puberty. We conducted high-resolution structural MRI brain scanning in young girls who underwent testing for HPG axis reactivation determined by stimulated LH concentration. We used HPG+ to represent being positive for HPG axis reactivation and HPG- for being negative for HPG axis reactivation. We hypothesized that the girls whose HPG axis reactivation was positive (HPG+) would show cortical thickness differences compared to the girls whose HPG axis reactivation was negative (HPG-), and there were associations between the cortical thickness of several regions showing differences between the two groups and the peak value of puberty-related hormone after the LHRH stimulation test in the entire sample.

## Methods

### Subjects

This study was approved by the ethics committee of the Second Affiliated Hospital and Yuying Children’s of Wenzhou Medical University. The LHRH stimulation test is an invasive procedure; it needs the intravenous injection of the medication and the collection of the blood sample three times in the test. Therefore, it is difficult to obtain the consent of the guardians to allow healthy girls to take it. We chose to look at these younger girls with thelarche whose bone age and uterine volume were advanced than their chronologic age as study participants. The positive for HPG axis reactivation was diagnosed premature puberty. The girls with premature puberty were recommended to non-clinical intervention such as exercise more and pay attention to their diets and so on and clinical following-up by doctor (6). Their parents also wanted to know their children’s height development thereafter. Because of the HPG-axis reactivation, the process is followed by the activation of the growth axis leading to growth spurts in puberty.

All the subjects provided written informed consent from the guardians before participation. One hundred five right-handed girls aged 8–11 years were recruited *via* the Child Healthcare Department of the Second Affiliated Hospital and Yuying Children’s of Wenzhou Medical University. The exclusion criteria were as follows: (1) intelligence quotient (IQ) < 70 estimated by the Chinese Wechsler Intelligence Scale for Children (C-WISC) (17), (2) history of premature birth or postterm birth, (3) history of neurological or psychiatric disorders in the participants or their first-degree relatives, (4) history of head trauma, (5) have any systemic physical illness, (6) any medications known to affect brain function or hormone levels, (7) menstruation, and (8) contraindications to MRI scanning. Finally, 105 girls were recruited: 63 were in the HPG+ group, and the others were in the HPG group. Details of the demographics of all subjects are shown in **Table 1**.

**Table 1:** Demographic and clinical characteristics of girls.

Characteristic		HPG+ (n = 63)		HPG- (n = 42)		*p*
		Mean	SD	Mean	SD	
Age (years)		9.13	0.57	8.92	0.36	0.064
BMI (kg/m^2^)		17.08	2.16	16.21	1.66	0.071
LH(IU/L)	LH max	21.09	15.60	3.36	0.86	<0.000
	LH baseline	1.04	1.197	0.117	0.065	<0.000
FSH(IU/L)	FSH max	16.59	6.53	12.94	5.13	<0.001
	FSH baseline	4.67	5.06	2.18	0.88	0.0022
E2(pg/ml)	E2max	39.44	15.43	32.45	14.04	<0.010
	E2 baseline	37.84	16.17	31.25	14.25	0.043
CBCL	Total score	10.52	10.64	12	10.51	0.156
IQ		91.11	14.50	89.79	12.05	0.12

SD, standard deviation; BMI, body mass index (BMI, calculated as Weight/Height^2^); HPG+/HPG-, with/without reactivated the HPG-axis; LH, luteinizing hormone; FSH, follicle stimulating hormone; E2, estradiol; TES, testosterone; CBCL, Child Behavior Checklist; IQ, intelligence quotient. p < 0.05 was considered statistically significant in all the tests.

### Assessment Scales and Pubertal Assessment

Intelligence tests were performed by means of Wechsler Young Children Scales of Intelligence (C-WISC) in all the subjects to screen for mental retardation before the MRI scanning. Then, assisted by a child care physician, the primary caretaker of each child completed the child behavior checklist (CBCL) (26), which is a self-report questionnaire used to assess the behavioral and emotional problems of the children. The details are shown in **Table 1**. Tanner staging was used to examine pubertal status. Mean ages and the standard deviations, the frequencies for Tanner breast stage and hair stage are showed in **Table 2**.

**Table 2:** Tanner stage.

Tanner stage	Tanner breast (mean age ± SD; frequency)		Tanner hair (mean age ± SD; frequency)	
	HPG+	HPG-	HPG+	HPG-
1	0	0	9.13 ± 0.57 63	8.92 ± 0.36 42
2	9.13 ± 0.57 63	8.92 ± 0.36 42	0	0

Mean age, standard deviations (in years), and frequencies for Tanner breast and hair stages.

Tanner breast 1: preadolescent, 2: appearance of the breast bud. Tanner hair 1: no public hair.

### LHRH Stimulation Test

All subjects received the LHRH stimulation test after the imaging data acquisition. Both exams were completed within a week. Following overnight fasting, the participants were told to arrive at the hospital at approximately 8:00 am. Four to five milliliters of blood were collected (0-min sample), and then the LHRH was injected as an intravenous bolus of 2.5 μg/kg (maximum dose < 100 μg) (27) *via* an indwelling catheter. At 30 and 60 min after injection, 2 ml of blood were drawn again.

The bloods samples from 0 min were sent immediately to the clinical laboratory for analysis, and the 30- and 60-min samples after the LHRH injection were sent immediately to analyze respectively. The samples were centrifuged, separated, and assayed. We assayed LH, FSH, and estradiol (E) using modern ultrasensitive automated chemiluminescence assays, and the lower limit of detection was 0.07 IU/L for LH, 0.3 IU/L for FSH, 10.7 pg/ml for E, and 0.1 ng/ml for TES separately. Intra-assay coefficient of variation (CV) was 3.72 and 3% at 31.98 and 63.02 IU/L for LH, 5.08 and 3.97% at 36.1 and 83.65 IU/L for FSH, 5.55 and 4.61% at 265.22 and 552.67 pg/ml for E, respectively. Inter-assay CV was 5.13 and 4.63% at 30.44 and 59.96 IU/L for LH, 5.58 and 5.07% at 36.68 and 83.91 IU/L for FSH, and 6.08 and 3.81% at 260.37 and 547.73 pg/ml for E, respectively. A serum LH level of ≥5 IU/L after the LHRH stimulation test from patient samples (either 30 or 60 min) is considered evidence of the positive for HPG-axis reactivation otherwise negative for HPG axis reactivation following previously established criteria (6–9). We used HPG+ to represent being positive for HPG axis reactivation and HPG- for being negative for HPG axis reactivation. The peak value of serum puberty-related hormone after the LHRH stimulation test (either 30min or 60min) was used for linear regression analysis. Details of the hormone levels are shown in **Table 1**.

### Structural Data Acquisition

The participants were scanned on a 3T GE HDxt scanner (General Electric, Milwaukee, Wisconsin) with an eight-channel phase array head coil. High-resolution T1-weighted images were acquired axially for the whole brain with a volumetric three-dimensional fast spoiled gradient recall (FSPGR) sequence. The scan parameters were as follows: TE/TR = 8.88 ms/4.02 ms, flip angle = 15°, FOV = 256 × 256, voxel size = 1 mm × 1 mm × 1 mm, and 160 slices with no gap. T2 FLAIR and T2-weighted images were acquired to screen the brain abnormalities by two experienced neuroradiologists. The quality of all the images was also controlled by them.

### Image Processing

The FreeSurfer package (version 5.30, http://surfer.nmr.mgh.harvard.edu/) was used for preprocessing of cortical thickness in high-resolution T1-weighted images. This method has high test-retest reliability (28, 29). The FreeSurfer pipeline performs motion correction on T1-images. The post-processing procedures have been explained in detail in previous studies (30, 31) and contained the following main steps: bias-field correction, Talairach and Tournoux atlas conversion, signal strength standardization, skull-stripped, automatically delineated gray/white matter boundaries, and the pial matter topology correction. Cortical thickness was calculated as the shortest distance between the white matter interface and the pial matter at each vertex across the cortical boundaries (31). These post-processing procedures were performed separately on each cerebral hemisphere.

### Statistical Analysis

Differences between HPG (+) and HPG (-) girls on demographic characteristics (e.g., age, BMI, CBCL total score, and IQ) and the hormone concentrations were examined using the independent sample Mann-Whitney U test.

The vertex-wise comparison of the cortical maps was performed on QDEC (Query, Design, Estimate, Contrast) with age as a covariate, and a whole-brain statistical threshold correction was performed using the Monte Carlo simulation method. Statistical significance was set at a cluster-wise–corrected P value < 0.05. A smoothing step using a Gaussian filter with 10-mm full-width at half maximum was initiated to average cortical thickness data across participants in a common spherical coordinate system.

Brain areas with statistical inter-group differences in cortical thickness were selected as brain region of interest (ROI). The average cortical thickness data of the ROI were extracted from the clusters. Correlation analysis between the regional cortical thickness values and stimulated hormone levels was assessed by using a general linear model and age as nuisance variables in the entire sample. Statistical significance was set at alpha < 0.05.

## Results

### Demographic Statistics

No statistically significant differences were found in the age (p = 0.064, >0.05), BMI (p = 0.071, >0.05), CBCL total score (p = 0.156, >0.05), IQ (p = 0.12, >0.05), and Tanner breast stage and hair stage between the HPG+ girls and HPG- girls. There were statistically significant differences in the levels of LH (p < 0.0001, <0.05), FSH (p = 0.0030, <0.05), and E (p = 0.0204, <0.05) between the two groups (**Table 1**).

### Group Differences in Cortical Thickness

**Figure 1:**
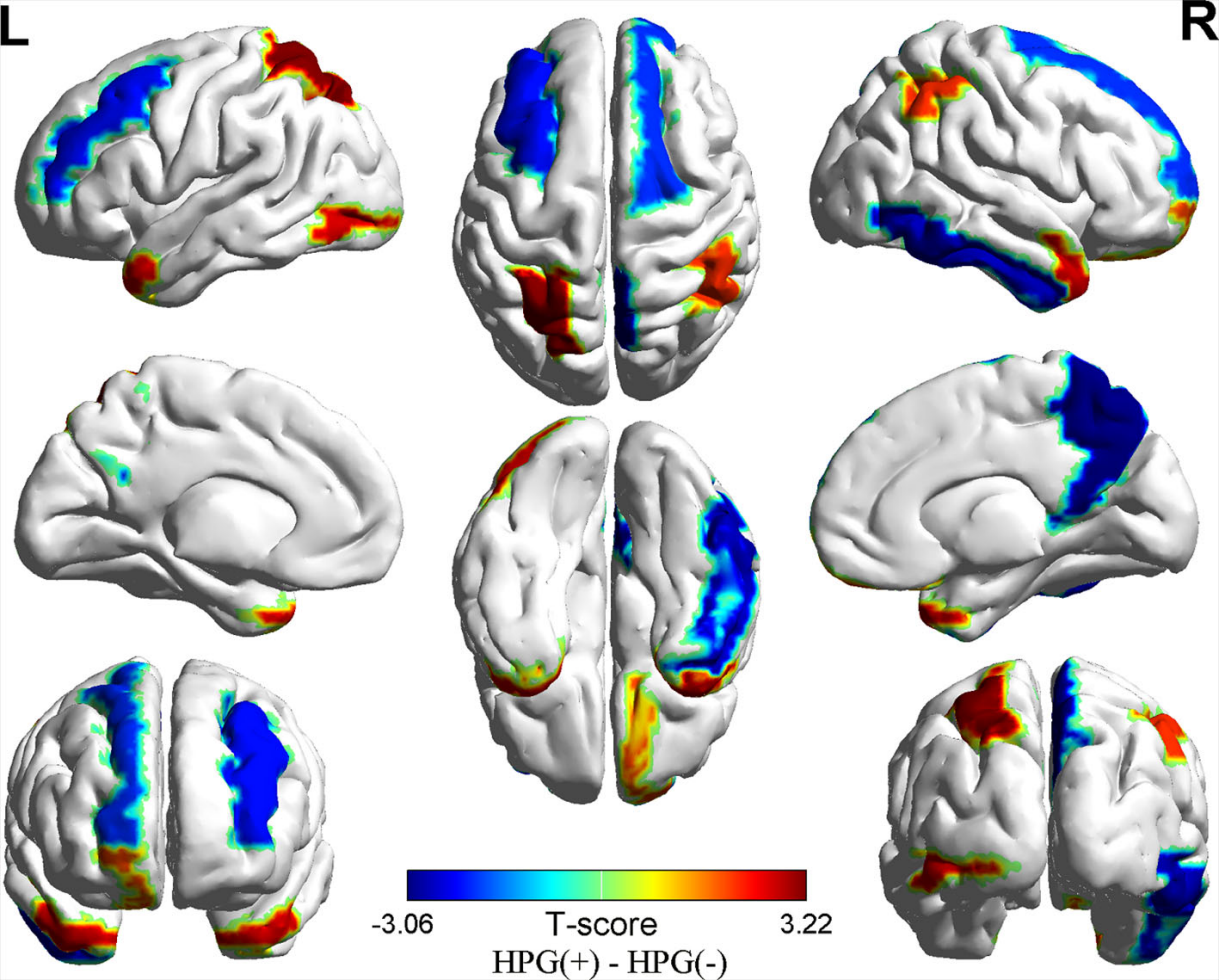
Differences in cortical thickness between the HPG+ girls and the HPG- girls. Decreased cortical thickness colored by blue and increased cortical thickness colored by red. HPG+ girls show decreased cortical thickness in the left rostral middle frontal gyrus, right precuneus, right inferior temporal gyrus, right superior frontal gyrus and increased cortical thickness in the right temporal pole, right inferior parietal lobe, right rostral middle frontal gyrusin, left superior parietal lobe, left lateral occipital gyrus, left superior temporal gyrus compared to the HPG- girls. Threshold at *p* < 0.05.

Compared to HPG(-) girls, it showed difference in cortical thickness of 10 brain regions in HPG(+) girls, less cortical thickness primarily in the right precuneus (p = 0.002, <0.05), right inferior temporal gyrus (p = 0.006, <0.05), right superior frontal gyrus (p = 0.044, <0.05) and in the left rostral middle frontal gyrus (p = 0.028, <0.05), and higher cortical thickness in the right temporal pole (p = 0.006, <0.05), right inferior parietal lobe (p = 0.003, <0.05), right rostral middle frontal gyrus (p = 0.04, <0.05) and in the left superior parietal lobe (p = 0.001, <0.05), lateral occipital gyrus (p = 0.009, <0.05), and superior temporal gyrus (p = 0.01, <0.05) (**Figure 1** and **Table 3**)

**Table 3:** Brain regions with significantly differences in cortical thickness between HPG+ and HPG-.

**Brain region HPG+ and HPG-**	**Cluster size (mm^2^)**	**MNI coordinates**			***P***
		X	Y	Z	
**Less**					
**Precuneus-R**	116.79	5.8	-65.1	34.3	0.002
**ITG-R**	70.18	48	-43.9	-16.3	0.006
**SFG-R**	23.91	10.2	0.8	51.7	0.044
**rMFG-L**	24.30	-28.2	32.0	30.7	0.028
**Higher**					
**Temporalpole-R**	105.91	33.1	14.3	-35.5	0.006
**IPL-R**	53.85	47.1	-54.4	44.6	0.003
**rMFG-R**	30.43	19.0	55.8	-13.9	0.04
**SPL-L**	61.69	-21.4	-51.6	62.1	0.001
**LOG-L**	184.07	-35.4	-82.5	-14.9	0.009
**STG-L**	55.43	-57.7	-43.2	15.8	0.01

Less, cortical thickness less in the HPG+ than in the HPG-; higher, cortical thickness higher in the HPG+ than in the HPG-; ITG-R, right inferior temporal gyrus; SFG-R, right superior frontal gyrus; rMFG-L, left rostral middle frontal gyrus; IPL-R, right inferior parietal lobe; rMFG-R, right rostral middle frontal gyrus; SPL-L, left superior parietal lobe; LOG-L, left lateral occipital gyrus; STG-L, left superior temporal gyrus.

### Associations Between Hormone Levels and Cortical Thickness in the Entire Sample

**Figure 2:**
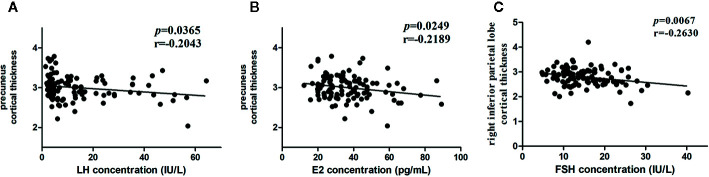
Associations between hormone levels and cortical thickness. **(A)** Negative correlation between the LH level and the cortical thickness in the right precuneus. **(B)** Negative correlation between the E levels and the cortical thickness in the right precuneus. **(C)** Negative correlation between the FSH levels and the cortical thickness in the right inferior parietal lobe.

We conducted a linear regression analysis to detect hormone-related variation in the cortical thickness of the differential brain region (10 ROIs) in the two groups. The results showed that the peak value of FSH after the LHRH stimulation test was negatively correlated to cortical thickness in the right inferior parietal lobe (r = -0.2630, p = 0.0067, <0.05), the peak value of LH and E were negatively correlation with cortical thickness in the right precuneus (r = -0.2043, p = 0.0365, <0.05; r = -0.2189, p = 0.0249, <0.05) (**Figure 2**). After the FDR correction, the results showed that the peak values of FSH after the LHRH stimulation test were negatively correlated to cortical thickness in the right inferior parietal lobe (p = 0.0403, <0.05).

## Discussion

To our knowledge, this study is the first to investigate the impact of HPG axis reactivation defined by LHRH stimulation testing on cortical thickness. Our main findings were (1) HPG+ girls who showed reduced cortical thickness mainly in the right precuneus, right inferior temporal gyrus, right superior frontal gyrus, and the left rostral middle frontal gyrus while increased cortical thickness primarily in the left superior parietal lobe and right inferior parietal lobe, the right temporal pole, right rostral middle frontal gyrus, left lateral occipital gyrus, and left superior temporal gyrus. (2) The correlation analysis revealed negative correlations between the peak value of LH and E after LHRH stimulation test and cortical thickness in the right precuneus, the peak value of FSH, and the right inferior parietal lobe in the two groups. Our results suggest that the reactivation of the HPG axis and related hormonal maturation may be involved in sculpting human brain cortical architecture.

One of the main interesting findings is the bidirectional changes of cortical thickness. In fact, previous studies indicated changes in the gray matter during different stage of puberty. For example, Herting et al. reported greater increases in Tanner stage predicted decreased cortical thickness including the right superior frontal gyrus and right superior temporal gyrus in puberty girls (15). While in a large group of 9-year-old twins, including males, the children that showed any signs of physical maturation (Tanner stage ≥ 2) was associated with decreased parietal gray matter densities compared to the children without any secondary pubertal signs (Tanner stage ≤ 1) (17). No studies have examined the difference in cortical thickness between prepuberty girls and girls of the same age at the onset of puberty. Our findings suggested that the reactivation of the HPG axis played a different role in the cortical thickness. The reactivation of the HPG axis drives dramatic changes in physical appearance, such as facial structure and the appearance of secondary sexual characteristics, and adolescents enter a stage of profound psychological transition in the onset of puberty, which may negatively or positively affect brain maturation. To reach a stable adult role, there are changes in the structure and function of the brain. Furthermore, multiple studies showed the receptors for HPG axis hormones are found in various brain cortical especially for learning and memory (32, 33). Most brain areas in our result were involved in learning and/or memory function. The reactivation of the HPG axis caused a cascade secretion of hormone, which attached to the receptors in the brain cortical. It might be one mechanism that the reactivation of the HPG axis sculpts the brain cortical thickness in our result.

Another important result demonstrates the correlations between the gray matter changes and the level of hormones. Sex steroids are known to affect the brain in two different ways: organizational effects and activational effects. The organizational effects of sex hormones were played by permanently changing the structure of the brain, such as neuronal number, myelination, and dendritic branching (34). Previous animal studies found that E levels could inhibit cortical myelination during puberty (35), and it is also involved in apoptotic processes (36). Another study found that estradiol altered dendritic spine density and dendritic spines in brain regions related to cognition and memory were sensitive to estrogen fluctuations (37). The precuneus was deemed to play a central role in a wide spectrum of highly integrated tasks, including self-centered mental imagery strategies, consciousness, and episodic memory retrieval (38). Yates and Juraska found that puberty ovarian hormone exposure reduced the number of myelinated axons in the splenium of corpus callosum in the rats (18). Koss and the colleagues also found that the number of neurons and glia were increased in the medial prefrontal cortex of female gonadectomy before puberty rats (20). Therefore, we hypothesize that higher estradiol levels might contribute to changes in the cortical thickness of the precuneus by decreasing the cortical number of neurons and/or myelinated axons.

Previous animal and human studies have shown that LH and FSH receptors have been found in various brain areas including the parietal cortex (39, 40). However, how circulating LH and FSH levels exert their effects on cerebral gray matter remains unclear. Bukovsky and colleagues speculated that microglial cells (among cells of the mononuclear phagocyte system) and some nerve cells in the gray matter of the human brain cortex might be influenced by LH. High LH levels might also activate resident macrophages to remove some nerve cells in which the expression of LH receptors was weaker to change gray matter (40). More studies are needed to further investigate the mechanisms. With regard to the relationship between the FSH level and gray matter, previous study showed that the gray matter volume of the right inferior parietal gyrus was decreased in perimenopausal women than premenopausal women (41). As we all know, the serum FSH level in perimenopausal women was obviously increased than in premenopausal. Another study found that the genetic variant for an FSH receptor gene strongly affected the onset of puberty in girls (42).

However, in contrast to our study, previous study of adolescence with an age of 8–25 years reported no effects of sex steroids on cortical thickness (12) and no association between the LH concentrations and gray matter (12, 24, 25). The possible explanations might be that (1) the hormonal level was determined in morning saliva or urine samples in previous studies and (2) the age of the sample. In that study the subjects were between 8 and 25 years. (3) The period the participants enrolled in [before or after hormonal maturation; middle or late puberty (23)] and the handling of confounding factors [stratification by sex or time in menstrual cycle (24)]. However, the onset age of puberty was between 7 and 13 in the girls, and the mean age of menarche was approximately 12 years old (43). To eliminate the effect of the menstrual cycle, the age of the participants was limited to 8–11 years old. All the subjects in our study were girls, none of them had menstruation, and their LH and FSH concentrations were peak-stimulated serum LH and FSH levels after the LHRH stimulation test.

The limitation regarding our study should also be noted. First, the age range of the sample is narrow. The age of the HPG (+) girls was 8 to 11 years, and that of the HPG (-) girls was 8 to 10 years. However, the onset age of puberty was between 7 and 13 in the girls, and the mean age of menarche was approximately 12 years old (43). Hence, to eliminate the effect of the menstrual cycle, the age of the participants was limited to 8 to 11 years old. Second, the serum concentration of LH, FSH, and E were the total hormone levels, encompass both bound and unbound hormone levels. Its precision to analysis the correlation between the puberty-related hormone and the brain regions used the unbound hormone levels. Thirdly, the current study was unable to detect more robust effects of the reactivation of the HPG axis; as we acknowledged, our results didn’t pass the statistical threshold at recommended corrected significance levels, the findings need for replication given the possibility of false-positive results in larger cohorts.

Despite the limitations, it’s important to emphasize the originality of the results by investigate the association of HPG axis reactivation defined by LHRH stimulation testing with cortical thickness in girls at the stage of premenarche. The current results would be useful to study the cellular mechanisms about how the sexual hormone effect the brain gray matter development, especially in the default mode network and executive control network highly associated with psychoneurosis at puberty.

## Conclusion

The present study provides evidence that reactivation of the HPG axis is associated with the development of cortical thickness, especially involving the default mode network and executive control network, in puberty along with hormone-related levels. These findings provide evidence for a better understanding of the relevance of the HPG-axis with brain maturation. Future studies should combine behavioral and functional measurements to illuminate the structure–function-hormone relationship.

## Data Availability Statement

The datasets generated for this study are available on request to the corresponding author.

## Ethics Statement

The studies involving human participants were reviewed and approved by the ethics committee of the Second Affiliated Hospital and Yuying Children’s of Wenzhou Medical University. Written informed consent to participate in this study was provided by the participants’ legal guardian/next of kin.

## Author Contributions

ZY conceived the study and designed the protocol. YF, DY, LX, PL, YZ, LZ, and TC did the experiments. WZ, BT, BY, and XL conducted the statistical analyses. YF, WZ, BT, BY, and ZY interpreted study findings and contributed to developing the manuscript. YF wrote the first draft of the manuscript that was revised by all authors.

## Funding

This research was supported by Natural Science Foundation of Zhejiang Province (grant number LY19H180003), Zhejiang Medical Health Science and Technology Program (grant numbers 2017KY108 and 2017ZD024), and Wenzhou Municipal Sci-Tech Bureau (grant numbers Y20150291 and Y20190185).

## Conflict of Interest

The authors declare that the research was conducted in the absence of any commercial or financial relationships that could be construed as a potential conflict of interest.
